# Marking-based load balancing Weighted Clustering Framework for Mobile ADHOC Network

**DOI:** 10.1371/journal.pone.0360099

**Published:** 2026-10-15

**Authors:** Prashant Dixit, Sonia Setia, Ramesh Chandra Sahoo, Manish Sharma, Sameena Pathan

**Affiliations:** 1 School of Computer Science and Engineering, Galgotias University, Greater Noida, Uttar Pradesh, India; 2 Department of CSE, MRIIRS, Faridabad, India; 3 Department of Electrical, Electronics and Communication Engineering, Galgotias University, Greater Noida, Uttar Pradesh, India; 4 Manipal Institute of Technology, Manipal Academy of Higher Education, Manipal, Karnataka, India; Anurag University, INDIA

## Abstract

In a mobile ad-hoc network, it has been observed that the existing weighted clustering algorithm induces high computational overhead on the mobile nodes in the cluster-head election process and distributes the load among the cluster-heads unfairly. Also, the cluster maintenance cost is very high due to the expiration or movement of the cluster-head. The purpose of this work is to mitigate the weaknesses of the existing weight-based clustering algorithm. The proposed “Marking-based Load Balancing Weighted Clustering Framework for Mobile ADHOC Network (MLWCM)” marks the weak nodes before the beginning of the election process and spares them from massive weight calculation, thus saving the scarce resources of the network. The concept of inmate affiliation reduces the maintenance cost considerably. To ensure fair load distribution among the cluster-heads, a handling factor has been computed, which is the number of nodes that a cluster-head can handle based on its available battery power. Also, to minimize the maintenance cost, a new event called inmate affiliation has been introduced. The simulation outcome reveals that the proposed framework outperforms the existing weighted clustering algorithm. MLWCM produces healthier and more stable cluster-heads. The number of members is fairly assigned to cluster-heads. Inmate affiliation allows a displaced node to get attached to an available nearby cluster silently, without inducing clustering in the complete network, which results in lower maintenance costs. Obtained results: By the simulation, it has been revealed that the MLWCM yields healthy cluster-heads in terms of battery power at a low computational cost, hence prolonging the life of the network and enabling efficient routing in the mobile ad-hoc network. For the proposed framework, MLWCM, the average battery life of the cluster-head is 73.8%, whereas for the existing WCA, the average battery life is 41.8%; hence, MLWCM enhances the stability of the network.

## 1. Introduction

In wireless communication, mobile ad-hoc networks are very accommodating, where the setup of infrastructure, like a base station, is nearly impossible due to geographical or time constraints [1]. The typical applications of Mobile ad-hoc networks include scenarios such as mobile conferencing, disaster management, rescue operations, battlefield communication, smart dust, etc. [2,3]. An ad-hoc network is constituted by mutually cooperative, battery-driven mobile nodes. Communication in an ad-hoc network takes place in a multi-hop fashion. Every node in the network may act as a sender, receiver, and router. Information packet forwarding decisions are taken in real-time due to the dynamic topology of the network; therefore, the classic Bellman-Ford algorithm used in wired networks is unsuitable to find the path between source and destination. In a mobile ad-hoc network, routing is broadly characterized into two classes, namely hierarchical and flat. In a flat routing algorithm, every node in the network works on the same level. For route construction, control packets are flooded throughout the network. Therefore, the same control packet may arrive at a given node multiple times, which leads to the problem of looping, so it occupies the network resources redundantly, and it gets worse when the number of nodes increases. In addition, flat routing can be of two types: proactive (table-driven) and reactive routing (on demand). In proactive routing methods, each node keeps the path in its routing table (table-driven) to all remaining nodes in the network in advance (proactively) all the time. Proactiveness increases the control overhead at a node as well as at the network level because every node has to maintain the path even if it is not required. The advantage of pro-activeness is that it reduces the delay, as the path to any node is available on the fly. An example of proactive routing is DSDV [3]. On the contrary, in reactive routing, a route construction routine is invoked whenever it is necessary. It reduces the control overhead at the cost of delay for the first packet to arrive at the destination. An example of on-demand routing is AODV [4]. All the flat routing algorithms are characteristically non-scalable because, with the increasing number of nodes, control over the head rises exponentially [5]. In this regard, hierarchical routing is useful for the scalability of the network [6]. Hierarchical routing algorithms partition the network into logical-hierarchical subgroups called a cluster. In the hierarchical approach, nodes in the network need to have information about their local topology only (inside the cluster). For intra-cluster transmission, any simple proactive routing is applied, and reactive routing is applied for inter-cluster transmission. In this work, only cluster-based routing algorithms (especially weighted clustering) are focused on. In the following sections of this work, clustering in a mobile ad-hoc network is discussed.

In a mobile ad-hoc network, scalability is achieved by partitioning the network into geographical groups known as clusters [7,8]. In other words, clustering is a grouping of neighboring nodes (two nodes are said to be neighbours if they are in the transmission range of each other). In cluster-based routing algorithms, mainly three types of nodes are defined: cluster-head, doorway, and ordinary node [9–11].

During the setup of the cluster structure, a cluster-head is elected among the network nodes by a certain cluster-head election approach. Actually, a cluster-head election process is to partition the network. After winning the election, the cluster-head and its neighbors form the cluster, and eventually, the entire network gets partitioned into clusters. The cluster-head acts as a local controller and is responsible for medium access control in its cluster. The cluster-head keeps the status of its members and facilitates intra-cluster and inter-cluster communication. Doorway nodes are the members of a cluster that can hear (in transmission range) the neighboring cluster-head or its member. Doorway nodes act as an interface between neighboring clusters as they are responsible for forwarding outgoing traffic and receiving incoming traffic. Ordinary nodes are cluster members that are neither cluster-head nor doorway nodes. Whenever a member needs to transmit to another node, it transmits the packet to its cluster-head. The cluster-head then checks if the destination is in its member list. If yes, it will forward the packet directly to the destination, and if the destination is outside the cluster, then the cluster-head forwards the packet to its doorway nodes. Subsequently, doorway nodes forward the packet to a nearby cluster. This process continues until the destination node is found. Thereafter, the acknowledgement packet backtracks the reverse path, and the path gets established. In the network, the union of cluster-heads and doorway nodes makes the backbone for routing the data, called the dominating set. In the well-formed clustered network, every node is either a member of the dominating set or the neighbor of at least one member of the dominating set. The cluster structure is depicted in Fig 1.

**Fig 1:**
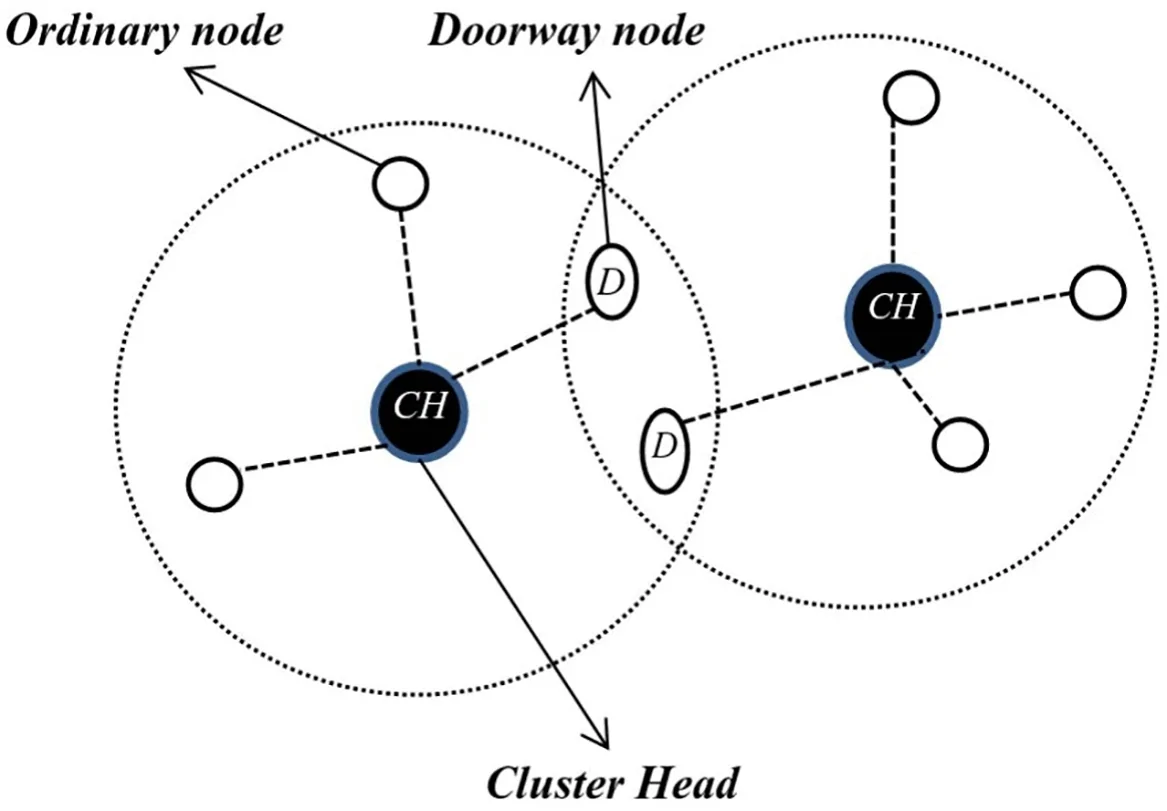
Standard Cluster structure.

It can be observed from the above discussion that clustering-based routing approaches have many recognizable advantages; one of them is the scaling of the network, which is increasing the number of mobile nodes. Clustering ensures this basic functionality with satisfactory performance [12,13]. Another one is the reusability of spatial resources like frequency or codes in non-overlapping clusters [14]. Another advantage is that the routing information exchange is limited to the backbone (cluster-heads and gateway nodes), which reduces traffic over the network [15]. Finally, nodes in the network need to be aware of their neighbors only (local topology); therefore, large and dynamic networks seem smaller and steadier in the view of every node [16,17]. Along with these advantages, clustering has consequences like an extra effort in creating and maintaining the cluster [18–20]. The efficiency of any clustering scheme is judged by the amount of effort required to set up the cluster structure [21–23]. In some clustering schemes, the occurrence of a local event like the termination or transit of a cluster-head may require the re-clustering of a complete network, which results in higher maintenance costs. Many researchers have put effort into improving clustering-based routing by minimizing the control overhead and ensuring a stable cluster structure [24–27].

## 2. Related work

The efficiency of a clustering-based routing principally depends upon its cluster-head election process. A clustering algorithm must identify the healthy (with sufficient power) and relatively motionless terminals to serve as cluster-heads. A clustering process should require less calculation and must be capable of maintaining the cluster structure at a reduced cost. Early cluster-head election approaches were simple and straightforward, for example, the lowest ID heuristic [28] and the highest degree heuristic [29].

The lowest ID heuristic [28] proposed that in the network, every single terminal is allotted a unique identifier (ID). At a consistent interval, every terminal announces its ID to its adjacent node, and every terminal compares its ID with its neighbour’s ID. A terminal with the lowest ID among its neighbours wins the election and becomes the cluster-head. This approach has the advantage that it is straightforward, thus requires less calculation and is easy to implement. But the disadvantage is that it may select a highly roving terminal as cluster-head, resulting in regular cluster breakage and re-clustering. The Highest degree heuristic [29] proposed that every node in the network calculates its degree (number of neighbours). Each terminal announces its degree in its neighbourhood. The terminal with the highest degree becomes the winner and acts as cluster-head. The advantage of this algorithm is that every cluster-head covers the maximum number of nodes. But there are numerous ties between nodes in the election process. Like the lowest ID heuristic, this algorithm may result in weak and roving cluster-heads. G. Chen et al combined the lowest Id and highest degree heuristic approach in k-CONID [30]. This algorithm is a generalization of the highest degree heuristic, as it allows the cluster membership up to *k* hops from the cluster-head, thus yielding multi-hop clusters. Every node in the network is assigned a duo called *did*= (*d, ID*), where *d* is the number of connections with nearby nodes up to k hops and *ID* is the unique identifier of the node. A node with the highest connectivity wins the election. If there is a tie between two nodes, the lowest ID heuristic breaks the tie, i.e., the node with the lowest ID becomes the cluster-head. K-CONID minimizes the number of clusters and lowers the size of the backbone. All these algorithms require periodic re-clustering to maintain the required attribute of cluster-head, which imposes high maintenance costs. To reduce this maintenance cost [31], proposed a least cluster change (LCC) in which cluster construction and maintenance are carried out in two different stages. Cluster construction follows the simple lowest Id heuristic, but re-clustering takes place in only two circumstances: when two cluster-head comes into the transmission range of each other, and second, if a node remains un-clustered, i.e., unable to listen to any cluster-head. LCC is a significant improvement over the highest degree and lowest ID algorithm, as cluster-head needs do not hold some specific attribute all the time, but the second case may still result in high maintenance costs due to the movement of a single node, which needs re-clustering of the entire network [32]. proposed an algorithm called 3-hop between adjacent cluster-head (3hBAC) in which the ripple effect of re-clustering is overcome by keeping the cluster-heads separate by 3 hops. Initially, one-hop clusters are created by following the highest degree heuristic. When two cluster-heads reach each other’s vicinity, any one of them has to relinquish its cluster-head status. In this way, neighbouring cluster-heads remain separated by 3 hops. In 3hBAC, a new status of the node called ‘cluster guest’ is presented. A node that is not a member of any cluster and cannot serve as a cluster-head, but can hear some member node, then attaches itself to that member node as a cluster guest. 3hBAC reduces the maintenance cost considerably, as re-clustering of the disturbed cluster does not raise the ripple effect in the entire network. Also, the concept of a guest node allows the un-clustered node to join the network silently, rather than create a separate cluster. But the cluster guest may overload the cluster-head. All these clustering algorithms overlook the mobility and lower battery power in all capacities of a node for being a cluster-head.

There is another category of clustering algorithms called collective weight-based clustering, which considers many factors such as remaining battery power, mobility, node degree, etc. The objective of collective weight-based clustering algorithms is to select the most capable node as cluster-head in its locality, rather than select based on parameters like lowest ID or highest degree. The main benefit of this type of algorithm is that it can tune the weighting factors for each weight to adapt according to different network circumstances. A standard example of this type of approach is the weighted clustering algorithm (WCA) [33]. Also, the conservation of energy for the IoT framework is discussed, where K-means clustering minimizes intra-cluster communication overhead [34,35]. WCA considers four factors; the first factor is the degree difference = | dv – δ |, where dv is the degree of the node, and δ is the ideal number of nodes that a cluster-head can handle efficiently. This parameter controls the load on the cluster-head, and the second parameter, Dv, is the sum of the distances from a node to all adjacent nodes. Dv indicates the energy requirement, as more energy is required for a larger distance. Third is Mv, which is the measurement of the mobility of a node; it is the displacement of node n for a specified time T. Fourth is Pv, which is the cumulative time for a node served as cluster-head. Pv indicates the power consumed as a cluster-head consumed more power than a non-cluster-head node. So the weight of the node is given by Wv = *w*1 + *w*2Dv + *w*3Mv + *w*4 Pv. Where *w1, w2, w3, and w4* are the weight factors for each parameter, such as the *w1 + w2 + w3 + w4* = 1. Initially, all nodes calculate their weight value and share it with their neighbours. The cluster-head will be the one with the least weight. This procedure continues until all nodes are members of some cluster.

The proposed work also belongs to the collective weight-based clustering category, which takes the WCA as a reference protocol. This work has identified the weaknesses of WCA and proposed solutions for them with a novel clustering framework, “Marking-based Load Balancing Weighted Clustering Framework for Mobile ADHOC Network”.

## 3. Gaps and proposed solution

In this work, the standard collective-weight-based clustering algorithm, the weighted clustering algorithm (WCA), has been studied, which reveals several intrinsic shortcomings. In this section, these shortcomings and their corresponding proposed remedies are discussed.

The foremost challenge in collective weight-based clustering algorithms is the large computation overhead. In the cluster-head selection process, every node has to compute its weight and exchange control messages in its locality for measuring the distance, speed, and degree, and for claiming the measured weight, which induces the communication overhead. Also, in the clustering process, every node loses scarce battery power in significant amounts, consequently reducing the network lifetime. In the proposed work “*Marking-based Load Balancing Weighted Clustering framework for Mobile ADHOC Network*” (MLWCM), mark the incompetent nodes whose possibility of winning the election is negligible since they don’t have a power level up to a predefined threshold, and spare them from participating in the election process. In this manner, the algorithm blocks the unnecessary injection of traffic and saves battery power, thus increasing the lifetime of the network.

Another weakness of the existing weighted clustering algorithm is unfair load distribution on cluster-heads. Because WCA takes the generalized model, the number of nodes δ that a cluster-head can handle, and based on δ, every node computes the degree difference by the formula **= | dv – δ |**. However, in a heterogeneous ad-hoc network environment, nodes are distributed randomly, so the degree of each node remains different for every node; therefore, the calculation of degree-difference with a common upper bound δ may leave many cluster-heads overburdened.

Also, the network may not be the same; therefore, the capacity to manage the number of nodes varies from node to node. In the proposed work, instead of the generalized upper bound, specified ***h*_**values are taken from a bucket called ***h***_table. ***h*_**value is defined as the ideal number of nodes that a cluster-head can handle w.r.t. its available battery power. The notion is that a node with more battery power can serve more nodes as a cluster-head efficiently for an acceptable time. ***h***_table contains the set of ***h_***values concerning the range of power level, such that Hi > Hi + 1 instance of ***h***_table is shown in Table I. There is a threshold of power level, below which the ***h***_value is set to zero, indicating that the node (having battery power less than a threshold) is not suitable for being a cluster-head and is spared from the election process.

Moreover, the available battery power (aliveness) of every node in the network. Furthermore, the values in the ***h***_table can be regulated according to the network scenario. For example, if the battery power is more crucial, then the tight bounds on power ranges may be kept; otherwise, it may be made flexible. In this work, the ‘Handling factor’ *Hv* is calculated by *Hv* = ***h***- *d(v)* instead of the degree difference. Here, the value of Hv is not considered *absolute* on purpose. The positive value of Hv reflects that the node has the number of neighbours under its capacity of being a cluster-head, and the negative value shows the number of neighbours is over the capacity (Table 1).

**Table 1:** Instance of *h*_table.

Range of power level	Ideal No node a cluster_head can handel(*h*_value)
100–80%	*h*_*1*_
79−65%	*h*_*2*_
64−40%	*h*_*3*_
39−20%	*h*_*4*_
<20%	**–**

The next shortcoming of the existing weighted clustering algorithm is identified as the estimation of the energy consumption of a node by the serving time of that node as a cluster-head. A cluster-head drains its energy proportionately to the amount of traffic that passes through it. In an ad-hoc network, as the traffic flows randomly, a newly elected cluster-head may pass more traffic than an older one. Therefore, the total serving time of being a cluster-head cannot guarantee energy consumption. Considering the above reason, in this work, precise outstanding battery power, Pv, is taken for calculating the competence value (weight). In the existing approach, a factor D*v* is the sum of the distances to all neighbours taken for weight calculation. But measuring the distance through receiving signal power is not the correct assessment of distance because a closer node transmitting a low-power signal may appear farther and vice versa. Moreover, the contribution of the sum of distances in weight calculation is not very significant because, in the broadcast system, a node merely needs to be in the transmitting range for communication. Another issue with the existing weighted clustering algorithm is that it allows only a one-hop cluster; that is, a member node can only be connected with its cluster-head through a direct link. This restriction leads to high maintenance costs. Because a small change in topology or the movement of the cluster-head may trigger re-clustering of the entire network. This phenomenon is called the ripple effect of re-clustering.

The limitation of cluster size up to the single hop creates a larger number of clusters than needed; consequently, the hop count over the path between the terminal will increase. To minimize the maintenance cost, the work done in *3-Hop Between Adjacent Cluster-head (*3hBAC) [32] is taken along with the use of and using a new position of node called an inmate node. Whenever a node is unable to get membership of any cluster, i.e., not in the transmission range of any cluster-head, but can hear a few member nodes of nearby clusters, then it can associate itself as *an inmate* node by one of these cluster members, as shown in Fig 2(a) and Fig 2(b). The inmate node is similar to the guest node in 3hBAC [32], but unlike in 3hBAC, in MLWCM the inmate node affiliates itself with the member node whose cluster-head has the largest handling factor, hence reducing the possibility for a cluster-head to get overburdened. The concept of an inmate node overcomes the ripple effect of re-clustering due to the movement or expiration of the cluster-head. If a member node is unable to hear its cluster-head, then it associates itself with the nearest cluster as a member or inmate node silently, rather than making a separate cluster, and causes the re-clustering of a complete network. Furthermore, the concept of inmate affiliation allows the merging of trivial clusters (clusters having only one node, the result of initial clustering) with primary clusters, consequently minimizing the number of clusters in the network. To ensure the separation of two clusters, if two clusters reach each other’s vicinity, then the cluster-head with a lower weight relinquishes its cluster-head status. This separation keeps the intra-cluster changes local.

**Fig 2:**
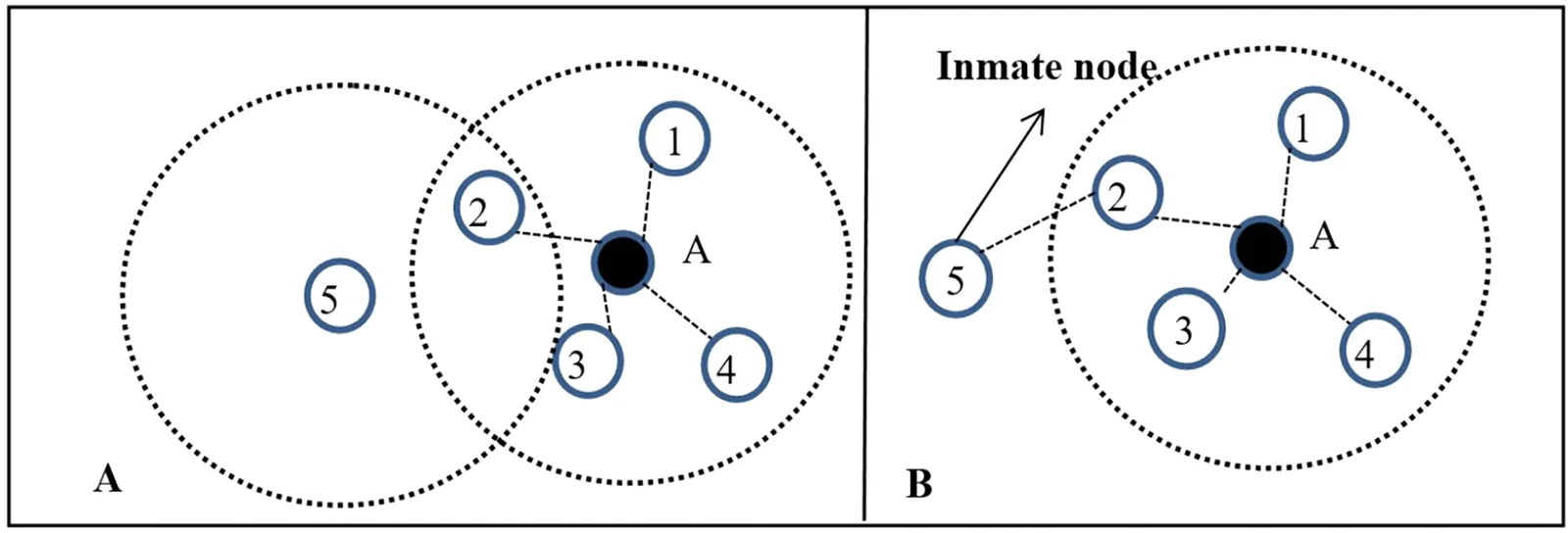
(a)-(b) Affiliation as inmate node.

## 4. Process

Based on the gaps identified in the preceding section, a proposed clustering framework, “Marking-based Load Balancing Weighted Clustering Framework for Mobile ADHOC Network” (MLWCM), is being presented.

### A. Basis and assumption for proposed work

The clustering algorithm performs in two phases: the first is initial clustering, and the second is the merging of trivial clusters (clusters containing only one node) with nearby clusters. During the formation of the cluster structure, the network topology is assumed to be static. Any mobile node can be a member of exactly one cluster.

After initial clustering, the cluster-head election procedure is invoked only when the dominating set is unable to cover all the nodes in the network. Initially, single-hop clusters are formed, and afterwards, clusters can extend up to two hops (the result of inmate affiliation or merging of trivial clusters). All nodes in the network possess a battery of the same capacity (mAh), and nodes can sense their outstanding power level. Nodes having power below a certain threshold are marked as weak and spared from the cluster-head election process. They can only be a member of a cluster. Every node in the network can measure its speed. All nodes in the network are aware of their suitable ***h*** value contained in ***h_***table for determining the handling factor H*v*. To elect the healthy cluster-head, MLWCM considers the following three parameters for the calculation of the competence value CF.

***Outstanding power P***_***v***_ ***(%):*** Outstanding power *Pv* is the assessment of the survival of a node and can be determined by Equation 1. *Pv* contributes to increasing the lifetime of the clustered network. A cluster-head with sufficient outstanding power increases the cluster’s existence and therefore reduces the maintenance cost due to the expiration of the cluster-head.


$$\begin{array}{c} P_{v}(\%)=\frac{presentpatterylevel(v)}{totalcapacity(v)}\text{1}\text{0}\text{0} \end{array}$$ (1)

***Handling factor H***_***v***_: Handling factor *H*_*v*_ is the assessment of how many member nodes a cluster-head can handle efficiently, considering its outstanding power level. *Hv* is the difference between the degree of a node *d(v)* (the algorithm for creating the neighbour set is shown in Fig 3), and its corresponding ***h*** value is calculated by Equation 2. *H*_*v*_ controls the size of the cluster for fair load distribution. A positive value of H_*v*_ indicates the number of members under the capacity, and a negative value is an alert of overcapacity.

**Fig 3:**
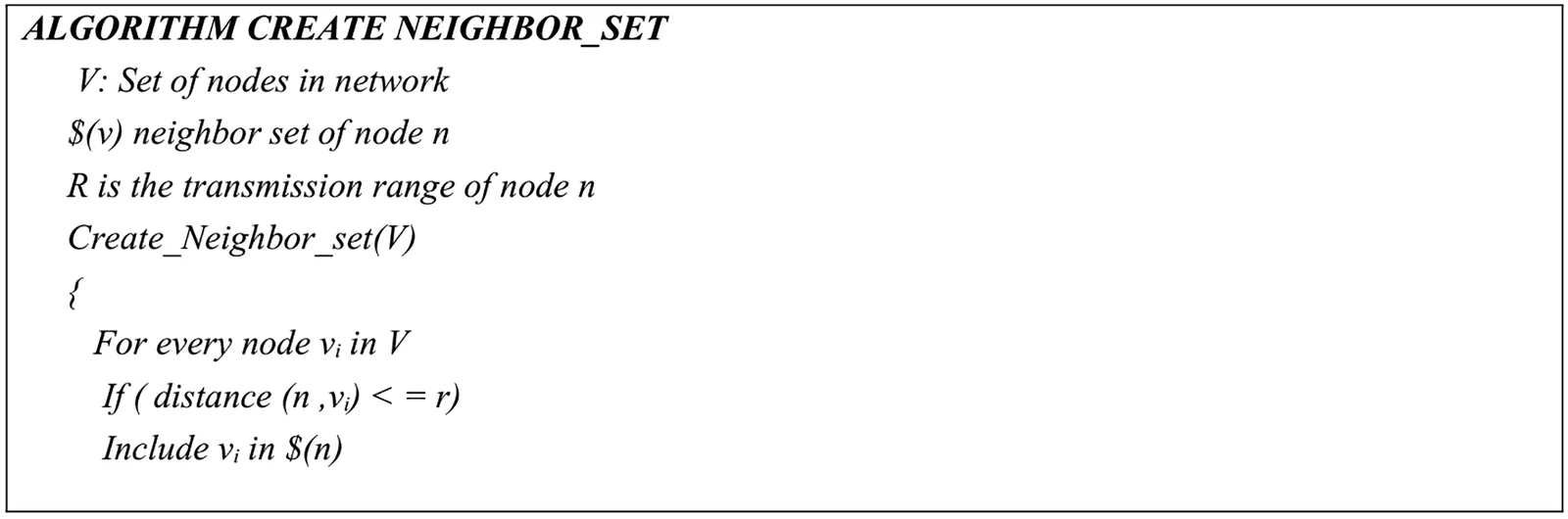
Algorithm creating a neighbor set.


$$\begin{array}{c} H_{v}=h-d(v) \end{array}$$ (2)

***Node average speed Sv***: Node speed is an assessment of the stability of a node. The average speed of a node can be determined by equation 3. A node with high mobility is inappropriate to play the cluster-head role as it leads to frequent cluster breakage and consequently higher maintenance costs.


$$\begin{array}{c} \text{Sv}=\frac{\text{1}}{\text{t}}\sum_{{\text{t}}_{\text{n}=\text{1}}}^{\text{t}}\sqrt{{\left({\text{x}}_{{\text{t}}_{\text{n }}}-{\text{x}}_{{\text{t}}_{\text{n}-\text{1}\;}}\right)}^{\text{2}}{-({\text{y}}_{{\text{t}}_{\text{n }}}-{\text{y}}_{{\text{t}}_{\text{n}-\text{1}\;}})}^{\text{2}}} \end{array}$$ (3)

Where *t* is the observed time and ($x_{t_{n\;},}{\text{x}}_{{\text{t}}_{\text{n }},}$ $y_{t_{n\;}}{\text{y}}_{{\text{t}}_{\text{n }}}$) and ($x_{t_{n-\text{1}\;}}{\text{x}}_{{\text{t}}_{\text{n}-\text{1}\;}}{\text{x}}_{{\text{t}}_{\text{n}-\text{1}\;}}$,$y_{t_{n-\text{1}\;}}{\text{y}}_{{\text{t}}_{\text{n}-\text{1}\;}}$) are the coordinates of node *V* at two consecutive time moments.

Clusterization and cluster-head election mechanism of the network are discussed in the next subsection.

### B. Clusterization of the network

System activation starts with the initialization of weighing factors α, β,γ, and the ***h***_table to meet the system requirements. For cluster formation, all the nodes in the network are assumed to know a common ***h***_table to determine their suitable ***h*** value corresponding to their outstanding battery level. Cluster formation is done in two phases: first, cluster-head election to form a 1-hop cluster, and second, merging of trivial clusters with nearby clusters formed in the first phase. The algorithm for phase I is shown in Fig 4.

**Fig 4:**
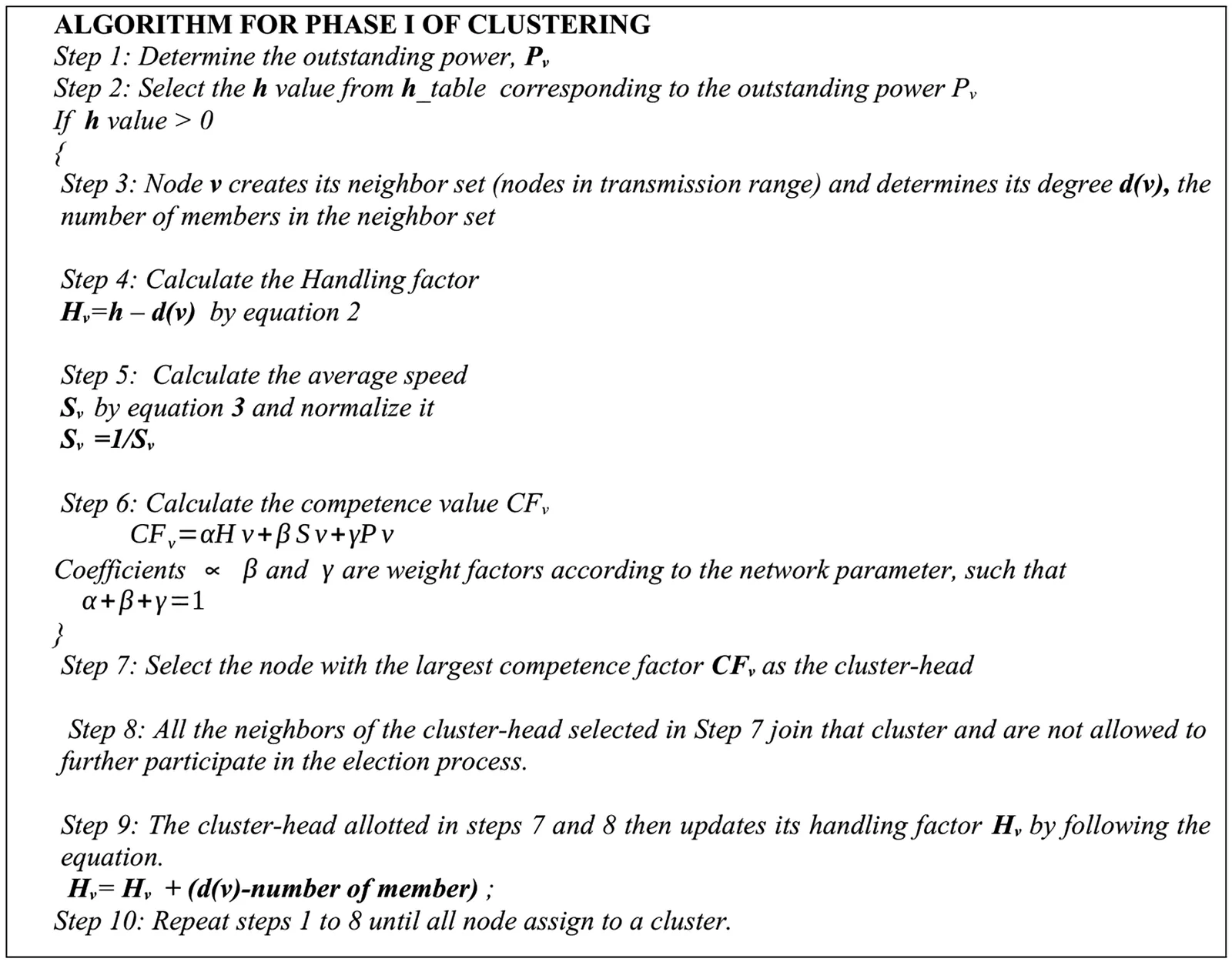
Algorithm for phase I of clustering.

In the second phase, cluster-heads determine if they have no member node; then they affiliate themselves with the nearby cluster by broadcasting the REQ_AFFI message (packet format for REQ_AFFI as shown in Fig 5. On receiving the REQ_AFFI message, neighboring nodes respond with the REPL_AFFI message containing their cluster-head ID CH_ID, along with the present handling factor **H**_***v***_ of its associated cluster-head. Thereafter, the requesting cluster-head associates itself with the member of the cluster-head with the largest handling factor by sending the INMATE message and changing its status to the inmate. Steps for Inmate affiliation are given in Fig 6. Fig 7 illustrates the packet format of the **REPL_AFFI (Reply Affiliation) message**, which is transmitted in response to a REQ_AFFI message during the second phase of clustering. The packet contains the unique identifier of the responding node (Node_ID), its associated cluster-head identifier (CH_ID), and the current handling factor of the cluster-head (Hv_of_CH). This information enables the requesting cluster-head to compare neighboring clusters and select the cluster-head having the highest available handling capacity, thereby promoting balanced member distribution and preventing cluster overloading. Fig 8 presents the packet format of the **INMATE message**, consisting of the Node_ID and Member_ID fields. After selecting the most suitable neighboring cluster based on the REPL_AFFI information, the requesting cluster-head transmits the INMATE message to confirm its affiliation with the selected cluster and changes its role to a member node. Thus, the INMATE message completes the affiliation process and facilitates the merging of trivial clusters, improving cluster stability and reducing unnecessary cluster-heads and associated maintenance overhead.

**Fig 5:**
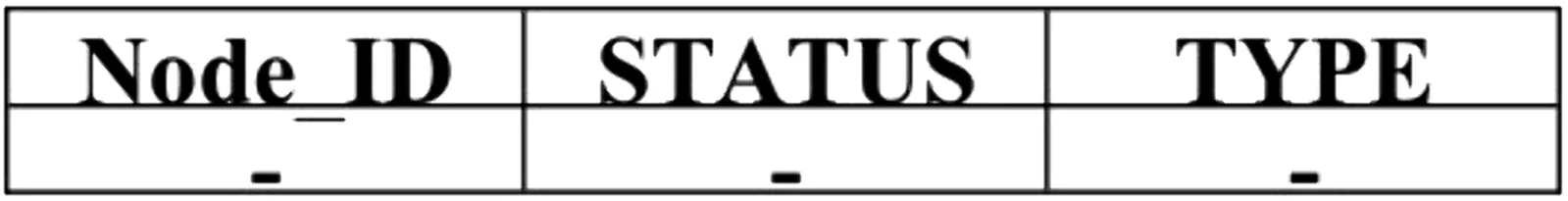
Packet format REQ_AFFI.

**Fig 6:**
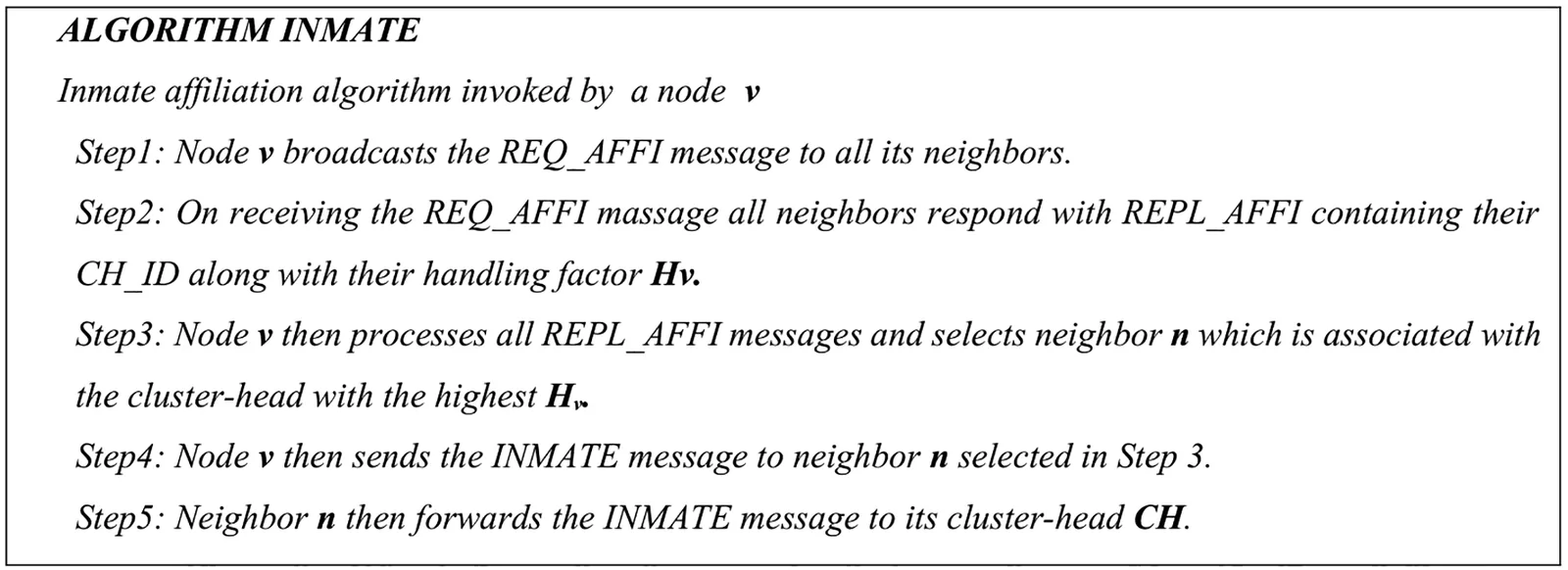
Algorithm for inmate affiliation.

**Fig 7:**
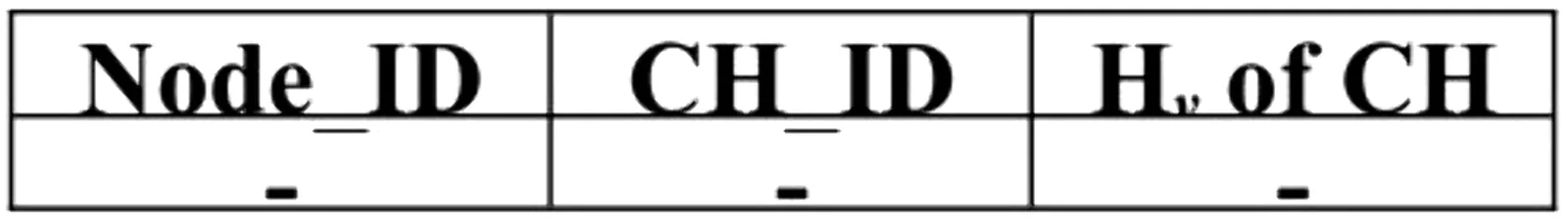
Packet format REPL_AFF.

**Fig 8:**
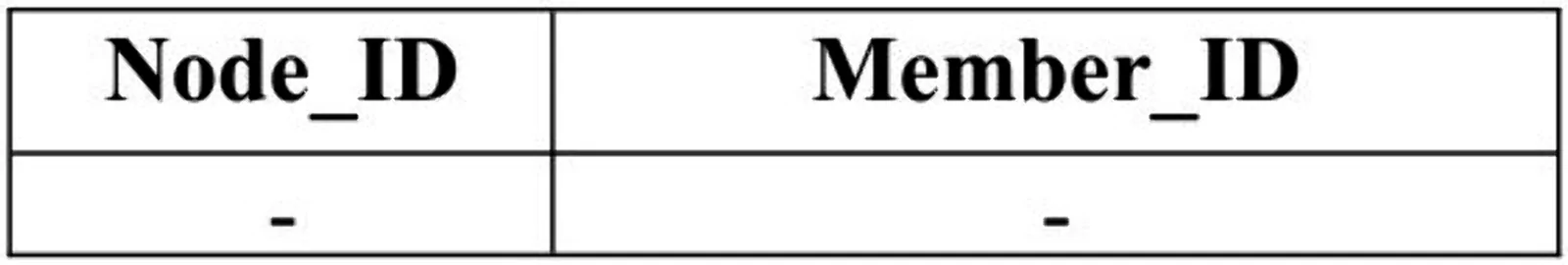
Packet format for INMATE message. Where: Node_ID: node’s unique ID, STATUS: CH, Gateway, member H_v__Of_CH: Handling factor of CH.

## 5. Results and discussion

Cluster formation by existing WCA and proposed clustering MLWCM has been simulated using MATLAB. Simulation has been performed on 40 randomly deployed mobile nodes, and they are labelled as 1–40. The area for simulation has been taken as 200 × 200 m^2^. For simplicity, the battery of each node is considered to be of the same capacity (mAh), and the outstanding battery power at every node varies from 1 to 100%. For the system activation of the proposed work MLWCM, ***h*** values in ***h_table*** are initialized according to the outstanding battery level, as shown in Table II. Cluster formation for both protocols is simulated in an identical network topology and node attributes. Simulation stages for both protocols, WCA and MLWCM, are presented in Fig 9(a), Fig 9(b), Fig 9(c), Fig 9(d), Fig 9(e), Fig 9(f), Fig 9(g), Fig 9(h), Fig 9(i), Fig 9(j), Fig 9(k) & Fig 9(l) and Fig 10(a), Fig 10(b), Fig 10(c), Fig 10(d), Fig 10(e), Fig 10(f), Fig 10(g), Fig 10(h), Fig 10(i), Fig 10(j), Fig 10(k) & Fig 10 respectively (Table 2).

**Table 2:** H_Table.

Range of power level	Ideal No. of node a cluster_head can handel (*h_value*)
100–80%	10
79−65%	8
64−40%	6
39−20%	4
<20%	0

**Fig 9:**
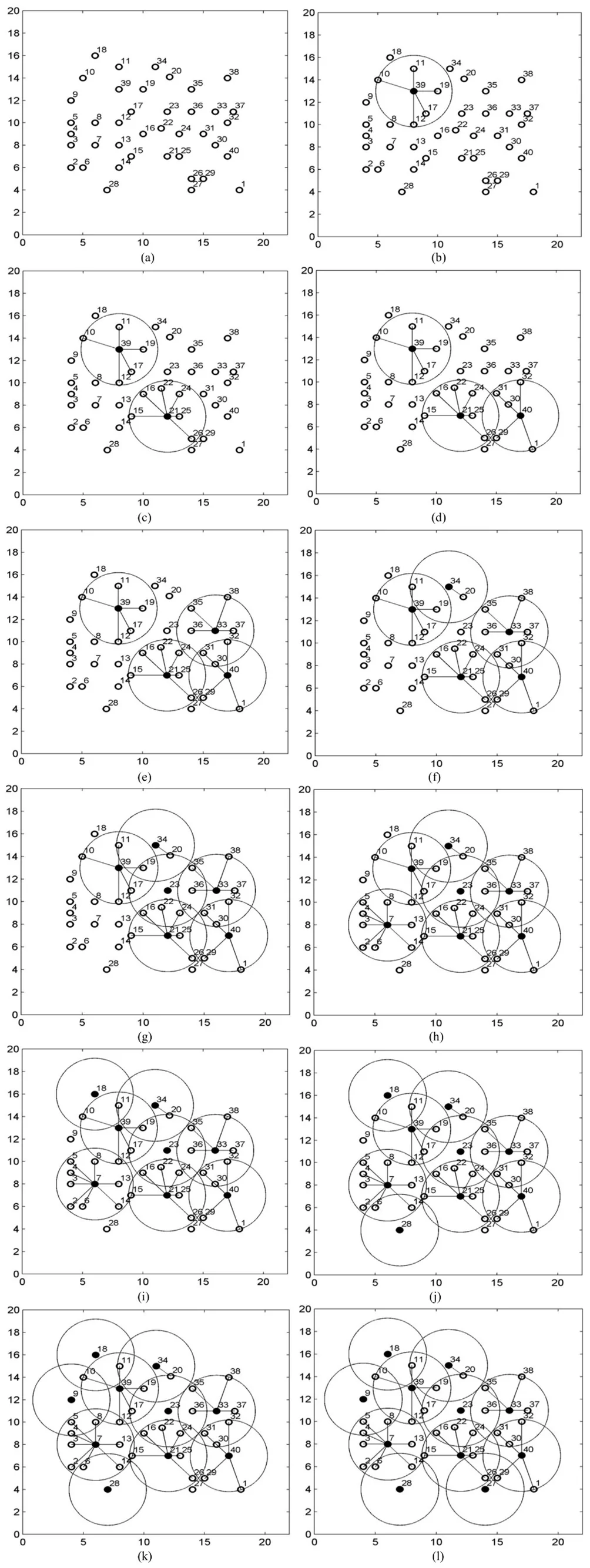
Findings of the cluster formation. (a) Initial topology of 40 nodes (b) Node 39 selected as cluster-head and made its cluster (c) Node 21 selected as cluster-head and made its cluster (d) Node 40 selected as cluster-head and made its cluster (e) Node 33 selected as cluster-head and made its cluster (f) Node 34 selected as cluster-head and made its cluster (g) Node 23 selected as cluster-head (h) Node 7 selected as cluster-head (i) Node 28 selected as cluster-head and made its cluster (j) Node 18 selected as cluster-head and made its cluster (k) Node 9 selected as cluster-head and made its cluster (l) Finally27 selected as cluster-head and made its cluster.

**Fig 10:**
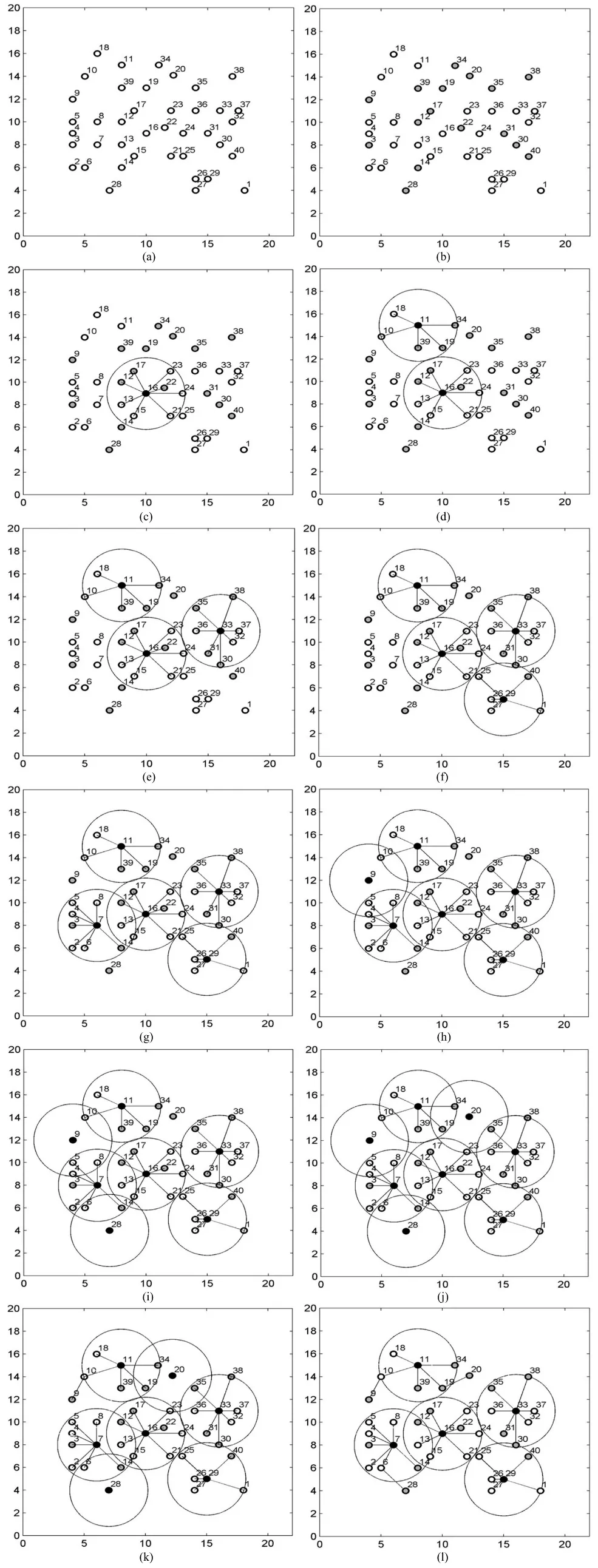
Topology with different nodes number. (a) Initial topology of 40 nodes (b) Marking of weak nodes (c) Node 16 selected as cluster-head and made its cluster (d) Node 11 selected as cluster-head and made its cluster (e) Node 33 selected as cluster-head and made its cluster (f) Node 29 selected as cluster-head and made its cluster (g) Node 7 selected as cluster-head and made its cluster (h) Node 9 selected as cluster-head and made its cluster (i) Node 28 selected as cluster-head and made its cluster (j) Node 20 selected as cluster-head and made its cluster (k). Node 9 merged with the nearby cluster as an inmate (l). Like node 9, node 28, and node 20 merged with nearby clusters and formed final clusters.

Simulation outcomes are tabulated and comprehended in Tables III, IV, and V. The simulation outcome of MLWCM has also been compared with the outcome of WCA and is presented in Table 4.

Simulation results show that in the case of existing WCA, the selected cluster-head is not healthy in terms of available battery life, which results in frequent cluster-head expiration and leads to higher maintenance costs. As the WCA allows a single-hop cluster, many uncovered single nodes form their own clusters, which results in more clusters than needed. Therefore, the hop count in the route between the communicating nodes increases.

Also, by taking the global ideal upper bound **δ**, the number of members in the clusters is unfairly distributed, which leaves some cluster-heads overloaded and some clusters underloaded, as shown in Table 3. For example, cluster-heads 39 and 40 have 5 members with only 16% and 12% battery life, respectively, whereas cluster-head 33 has 4 members with 67% battery life.

**Table 3:** Simulation results of Cluster Information using WCA.

S.N.	CH	Members	NO_OF Members	Degree Difference	Outstanding power	Mobility	Weight_Of CH
1	39	10,11,12,17,19	5	1	16	0.0100	1.4428
2	21	15,16,22,24,25, 26	6	0	64	0.0100	1.4472
3	40	1,29,30,31,32	5	1	12	0.0100	1.5263
4	33	35, 36,37,38	4	1	67	0.0100	1.8171
5	34	20	1	3	18	5.4500	2.9086
6	23	–	0	2	56	2.1200	3.0769
7	7	2,3, 4,5,6,8,13, 14	8	4	85	0.1000	3.3248
8	18	–	0	4	63	3.7100	3.5602
9	28	–	0	4	10	7.8300	3.6554
10	9	–	0	2	19	4.0000	5.8064
11	27	–	0	3	50	5.6100	22.8406

Moreover, WCA imposes huge calculation overhead at every node, even if the possibility of the node winning the election is negligible. In the case of MLWCM, selected cluster-heads are comparatively stable and healthier. The MLWCM concept of inmate affiliation allows the uncovered node to merge with a nearby cluster that yields a reasonable number of clusters.

By keeping the ideal cluster-head degree ***h_value*** according to outstanding battery life, the number of members per cluster is fairly allotted as shown in Fig 10(a)-Fig 10i and Table 4. Also, MLWCM identified 16 nodes out of 40 that are not suitable for the cluster-head role, hence sparing them from heavy-weight calculation.

**Table 4:** Simulation results of Phase-I of Cluster Formation using MLWCM.

S.N.	CH	MEMBERS	NO_OF Members	Handling factor	Outstanding power	Mobility	Competence Factor_Of CH
1	16	12,13,15,17, 21, 22,23,24	8	2	90	0.0100	66.6000
2	11	10,18,19,34 39	5	3	77	0.0100	61.7000
3	33	30,31, 32,35,36,37,38	7	1	67	0.0100	57.1000
4	29	1,25,26, 27,40	5	1	60	0.0100	54.0000
5	7	2,3,4,5, 6, 8, 14	7	1	85	0.1000	32.4000
6	9	–	0	−6	19	4.0000	4.6750
7	28	–	0	−8	10	7.8300	1.0383
8	20	–	0	−6	8	6.1800	0.2485

Simulation findings of the cluster formation by using the WCA are shown in Table 3.

Next, the simulation stages of cluster formation in MLWCM are shown in Fig 10(a), Fig 10(b), Fig 10(c), Fig 10(d), Fig 10(e), Fig 10(f), Fig 10(g), Fig 10(h) and Fig 10(i). Nodes have been assigned unique IDs from 1 to 40 randomly; the radio range of the node is shown by a bold circle shown around the node. A square area of 200 × 200 m^2^ has been considered for the distribution of the nodes.

Simulation findings of cluster formation in the case of MLWCM of phase I are shown in Table 4.

Simulation findings of cluster formation in the case of MLWCM of phase II are shown in Table 5.

**Table 5:** Simulation results of the Merging Phase of Cluster using MLWCM.

S.N.	CH	MEMBERS	NO_OF Members	Handling factor	Outstanding power	Mobility	Competence Factor_Of CH
1	16	12,13,15,17, 21,22,23,24	8	2	90	0.0100	66.6000
2	11	10,18,19,34 39,9,20	5	1	77	0.0100	61.7000
3	33	30,31, 32,35,36,37,38	7	1	67	0.0100	57.1000
4	29	1,25,26, 27,40	5	1	60	0.0100	54.0000
5	7	2,3,4,5, 6, 8, 14,28	7	0	75	0.1000	32.4000

A comparison of simulation findings of cluster formation using WCA and MLWCM has been made and presented in Table 6.

**Table 6:** Comparison of outcomes of WCA and MLWCM cluster formation.

WCA				MLWCM			
**CH**	**NO_OF** **Members**	**Outstanding** **Power %**	**Mobility**	**CH**	**NO_OF** **Members**	**Outstanding** **Power %**	**Mobility**
39	5	16	0.0100	16	8	90	0.0100
21	6	64	0.0100	11	5	77	0.0100
40	5	12	0.0100	33	7	67	0.0100
33	4	67	0.0100	29	5	60	0.0100
34	1	18	5.4500	7	7	75	0.1000
23	0	56	2.1200	–	–	–	–
7	8	85	0.1000	–	–	–	–
18	0	63	3.7100	–	–	–	–
28	0	10	7.8300	–	–	–	–
9	0	19	4.0000	–	–	–	–
27	0	50	5.6100	–	–	–	–

It can be comprehended from Table 4 & Table 5, and Fig 10(a), Fig 10(b), Fig 10(c), Fig 10(d), Fig 10(e), Fig 10(f), Fig 10(g), Fig 10(h), Fig 10(i), Fig 10(j), Fig 10(k) & Fig 10(l) that MLWCM generates 8 clusters of single hop in the first phase. Thereafter, trivial clusters containing only cluster-heads 9, 28, and 20 get merged as an inmate node with nearby clusters, considering their handling factors, and end up with only 5 well-formed 2-hop clusters, which reduce the hop count between the communicating terminals significantly.

Fig 11 represents the outstanding power level of elected cluster-heads in both WCA and MLWCM. It reveals that MLWCM elected the healthier cluster-heads (in terms of available battery power) comparatively, which prolongs the network life; for the persistence of the network, it is imperative to keep the cluster-heads alive for a maximum timespan, since the members of a cluster communicate through the cluster-head (inter- cluster or intra-cluster); therefore, the age of cluster-heads determine the life of network. Fig 11 also reveals that the number of clusters formed in the case of MLWCM is 5, which is much less than in the case of WCA, which is 11, implying less network overhead in the case of MLWCM; since clusters to maintain the network are fewer, control packet transmission is reduced, which increases the efficiency of the network in terms of battery power and congestion (every node in the network, processes the minimum control packets).

**Fig 11:**
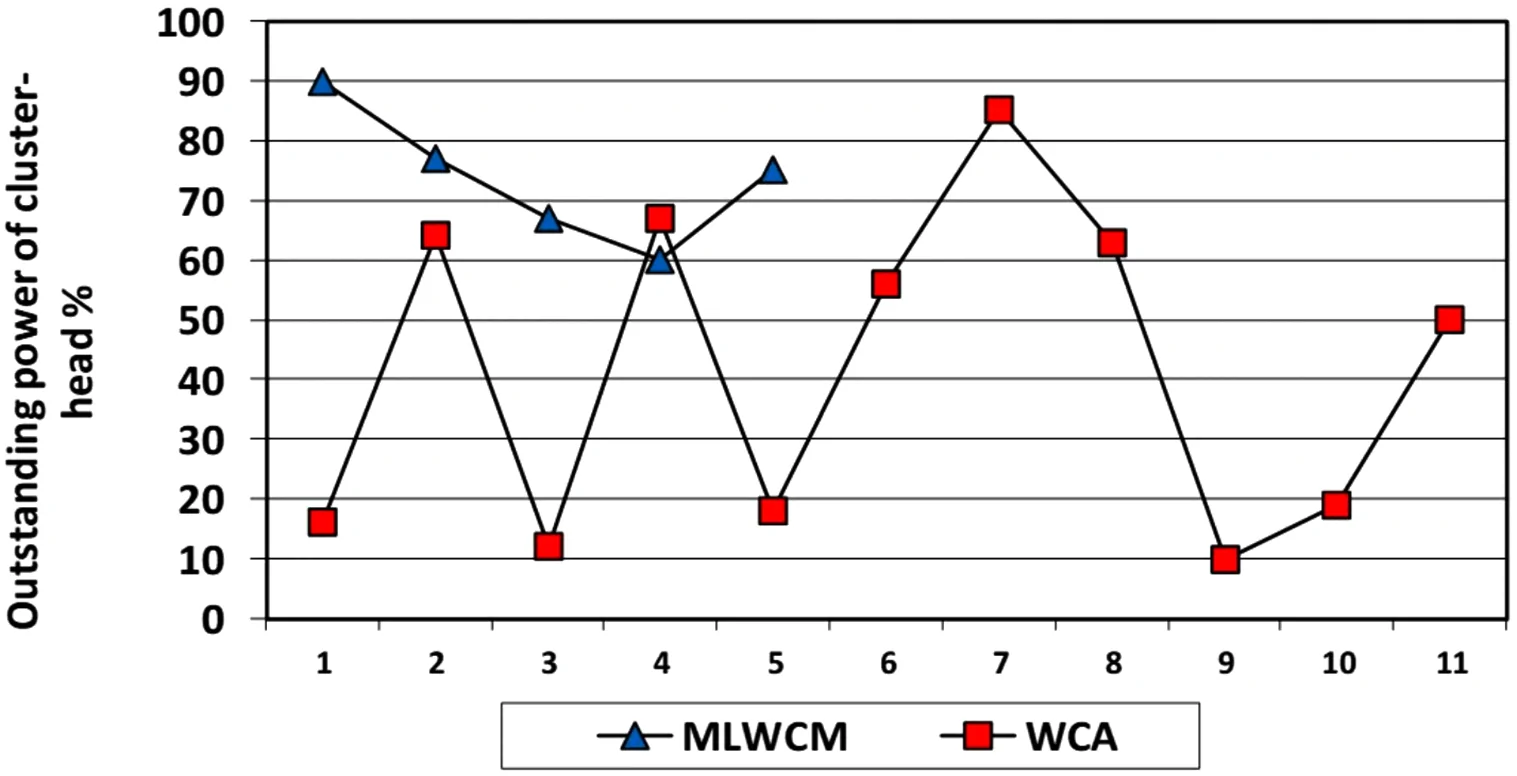
Outstanding power of cluster-head in WCA and MLWCM.

Fig 12 represents the mobility of elected cluster-heads. It also reveals that MLWCM elected a relatively stable cluster-head in terms of mobility, since the mobility of the cluster-head and the control overhead to the network are reciprocal; a stable cluster-head ensures the prolonged network life. In Fig 12, the triangle node represents MLWCM, and the square nodes represent WCA.

**Fig 12:**
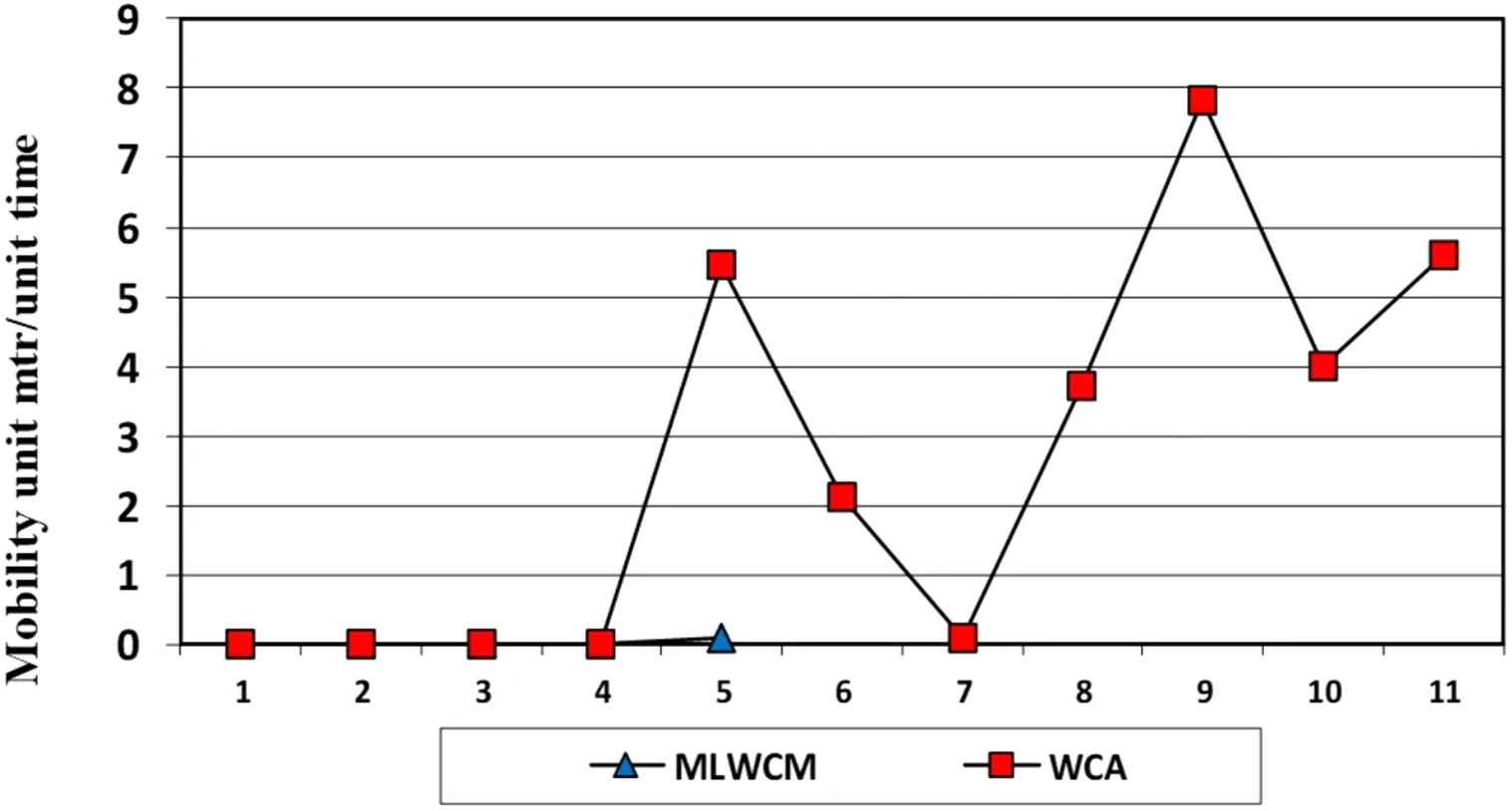
Mobility of cluster-head in WCA and MLWCM.

The distribution of members throughout the clusters with respect to the cluster-head outstanding power is comprehended in Fig 13 and Fig 14 for WCA and MLWCM, respectively.

**Fig 13:**
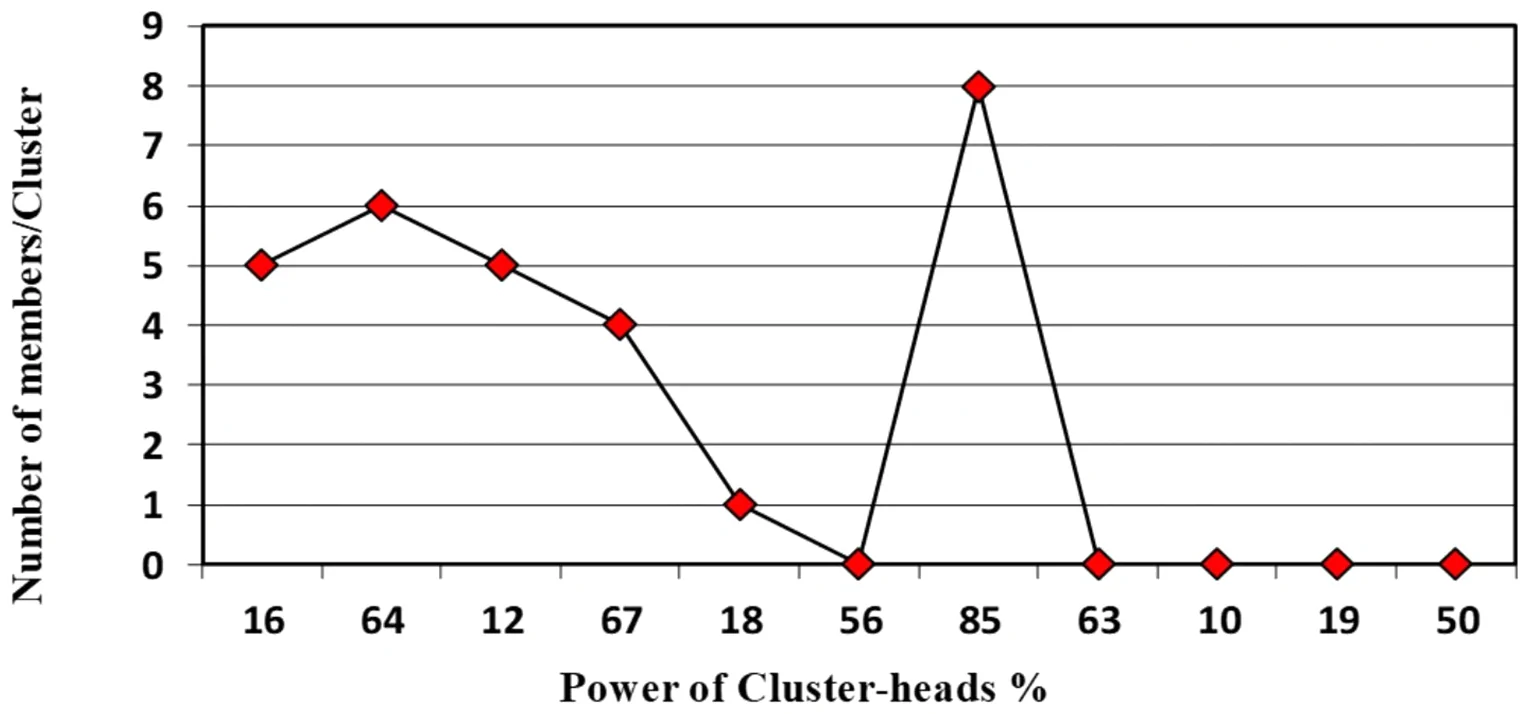
Member assigned vs. outstanding power of cluster-head in WCA.

**Fig 14:**
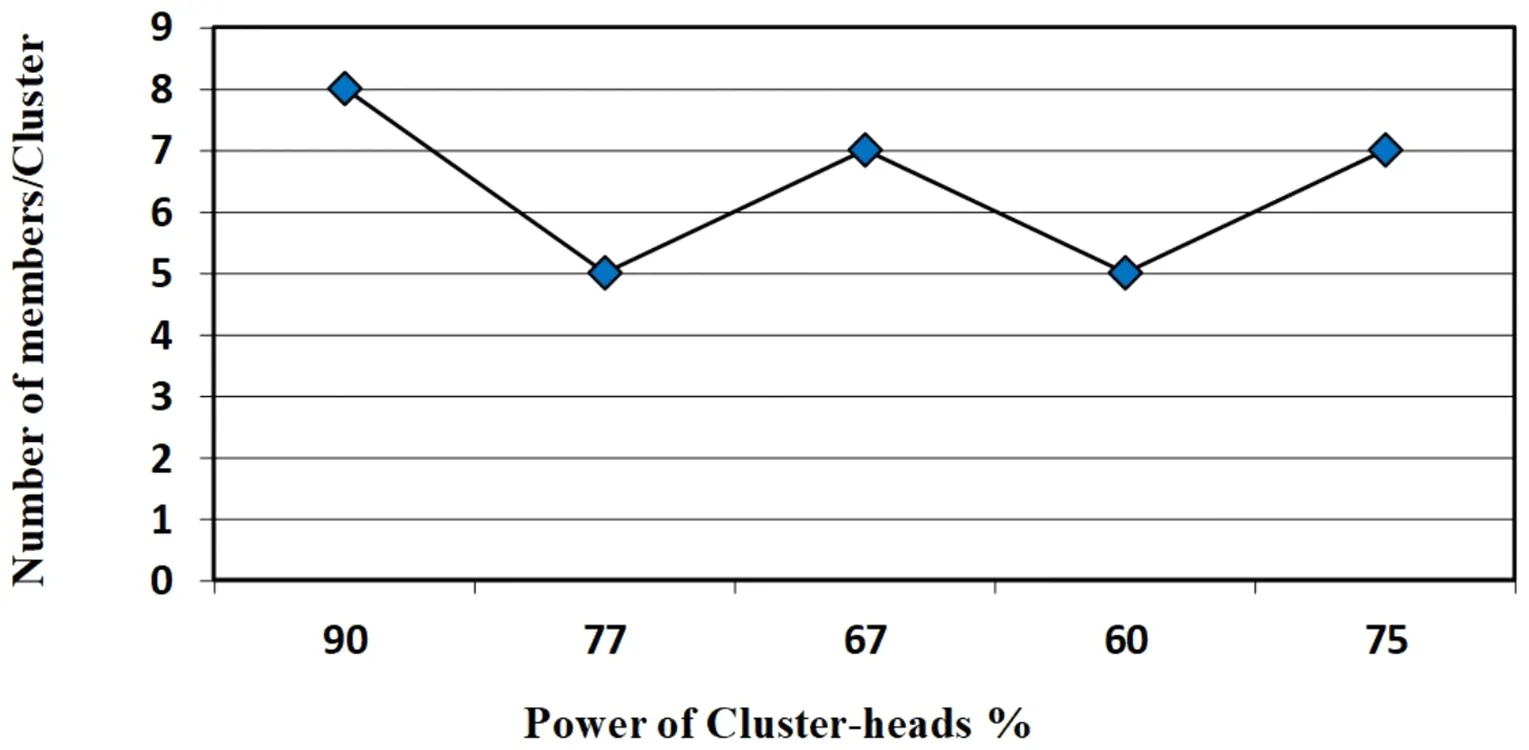
Member assigned vs. outstanding power of cluster-head in MLWCM.

It can be observed that the assignment of members to cluster-heads is not fair in the case of WCA, for instance, a cluster-head with remaining power 67% assigned 4 members and a cluster-head with outstanding power 16% is assigned 5 members, in another instance, a cluster-head with remaining power 12% assigned 5 members and a cluster-heads with outstanding power 50% and 63% are assigned no members at all, this unfaire distribution of member to cluster-head makes network fragile and less persitant as members will put the load on their cluster-head more than the capacity which leads to the frequent link breakage. The average battery power of the cluster-heads is 41%.

While in the case of MLWCM, members assigned to cluster-heads are almost uniformly distributed with respect to outstanding power; for instance, cluster-heads with outstanding power 90% assigned 8 members, and cluster-heads with outstanding power 60% assigned 5 members. The average battery power of the cluster-head is 73%.

## 6. Conclusion and future scope

In this work, standard cumulative weight-based cluster-head selection algorithms are studied in detail, and their inherent shortcomings are identified. To counter the gaps in existing algorithms, a novel clustering framework, “Marking-based Load balancing Weighted Clustering framework for Mobile Ad-hoc Networks” (MLWCM), is proposed. Simulation results reveal that the existing weighted clustering algorithm produces an unnecessary number of clusters with weaker cluster-heads. In the case of MLWCM, healthy cluster-heads are selected, and the concept of inmate affiliation yields a reasonable number of clusters to cover all nodes in the network. The existing weighted clustering algorithm imposes a high calculation overhead on the network and node levels. MLWCM marks the weak nodes before the beginning of the election algorithm and spares them from massive weight calculation and saves network resources. In existing WCA, the movement or termination of only one node can cause re-clustering in the complete network, which in turn results in higher maintenance costs. On the other hand, in MLWCM, inmate affiliation allows a displaced node to attach to an available nearby cluster silently, without triggering the re-clustering of the entire network. Also, in the existing WCA, the global ideal number of nodes **δ** for a cluster-head distributes the load among the cluster-heads unfairly. Whereas in MLWCM, a specific ***h***_value controls the load distribution over the clusters according to the outstanding battery level of cluster-heads. All in all, MLWCM partitions the network in a better way by selecting healthy and stable cluster-heads with lower maintenance costs.

The proposed framework identifies the stable cluster-head with a reasonably smaller number of clusters; however, a prediction model for marking vulnerable nodes in the network can omit the link breakage that lowers the maintenance cost of the network and enhances the stability of the cluster.

Researchers can go for a larger number of simulations with diverse network parameters, and also harness the research with statistical analysis, mean, variance, and confidence intervals. However, the proposed research has considered mobility; researchers can test the model in a highly dynamic topology with a larger number of nodes. The future scope of the proposed clustering framework also includes several important enhancements to improve robustness and real-world applicability. Further work can focus on conducting multiple simulation runs with different random seeds and applying statistical analysis such as mean, variance, and confidence intervals to strengthen empirical reliability. Evaluating the algorithm under dynamic network conditions with continuous node mobility and frequent topology changes would better represent practical scenarios. Scalability analysis for large-scale and high-density networks is also essential to assess computational efficiency and overhead. The framework may be extended by incorporating energy-aware and Quality of Service (QoS) metrics to improve cluster-head selection and network lifetime. Real-time or hardware-based implementations can validate feasibility beyond simulations. Additionally, integrating security mechanisms and intelligent optimization techniques such as machine learning–based adaptive parameter tuning could further enhance performance, adaptability, and resilience in heterogeneous networking environments.

Future work will also validate the proposed framework in larger-scale MANET environments with a substantially higher number of nodes and heterogeneous battery capacities. Further investigations will also consider diverse mobility patterns, traffic loads, and network densities to evaluate the scalability, robustness, and practical applicability of the proposed clustering approach.
